# Features of interconnection between temperament, self-esteem and aggressiveness in adolescents with mental and somatic pathology

**DOI:** 10.1192/j.eurpsy.2021.578

**Published:** 2021-08-13

**Authors:** N. Zvereva, M. Zvereva

**Affiliations:** Clinical Psychology, Federal State Budgetary Scientific Institution Mental Health Research Center, Moscow, Russian Federation

**Keywords:** aggressiveness, temperament, self-esteem, adolescents

## Abstract

**Introduction:**

Adolescence can manifest different in norm and in illness. It’s important to find common characteristics of adaptation with different types of ontogenesis, or leading manifestations of disease

**Objectives:**

Three adolescence (boys&girls) sample: normal – 22, middle age 16, cardio pathology – 7, middle age 16, psychopathology – 12, middle age 15

**Methods:**

Direct self-esteem by Dembo-Rubinstein (DR) test and indirect self-esteem by color attitude test by Etkind (CAT), Structure of Temperament Questionnaire (STQ-77), Buss-Perry Aggression Questionnaire (BPAQ).

**Results:**

Significant differences (criteria Kruskal–Wallis) were obtained on scales BRAQ “Hostility” (H= 8.430, p<0.015), “Common aggression” (H= 8.347, p<0.015), STQ-77 “Physical Endurance” (H= 9.895, p<0.007), “Physical Tempo” (H= 8.579, p<0.014), “Social Endurance” (H= 7.902, p<0.019), “Social Tempo” (H= 7.736, p<0.021), “Plasticity” (H= 7.797, p<0.020), “Self-confidence” (H= 7.157, p<0.028), “Neuroticism” (H= 8.179, p<0.017); gaps DR-CAT for scales “Health” (H= 12.330, p<0.002), “Happiness” (H= 7.296, p<0.026). Pearson correlation coefficient between STQ-77, BRAQ and Gaps DR-CAT found in normal group: Gap DR-CAT “Health” – STQ-77 “Physical Endurance” (r=-.508, p<0.05), Gap DR-CAT “Smart” - STQ-77 “Intellectual Endurance” (r=-.521, P<0.05), Gap DR-CAT “Happiness” – BRAQ “Hostility” (r=.528, p<0.05), Gap DR-CAT “Happiness” - STQ-77 “Impulsivity” (r=.432, p<0.05), “Neuroticism” (r=.539,p<0.01). Correlation was founded in cardio pathology group: Gap DR-CAT “Smart” – BRAQ “Physical aggression” (r=.857, p<0.05), “Anger” (r=.842,p<0.05), “Common aggression” (r=.860,p<0.05), Gap DR-CAT “Happiness” – BRAQ “Physical aggression” (r=.826,p<0.05), “Anger” (r=.773,p<0.05), “Common Aggression” (r=.787,p<0.05). For psychopathology wasn’t found correlations.

**Conclusions:**

Comparative study of personality traits of adolescents with different types of ontogenesis (normotypical, mental, cardio pathology) is important for evaluating their adaptation and determining targets of psychotherapeutic work.

